# Influence of stress and knowledge on self-medication practices among pharmacy students: a cross-sectional study

**DOI:** 10.3389/fmed.2026.1877740

**Published:** 2026-09-11

**Authors:** Eman O. Alsheikh, Nader N. Al-Majnoni, Majed A. Al-Zahrani, Abdullah S. Al-Saedi, Khalid A. Al-Marhabi, Danah K. Al-Areshi, Walaa S. Abu Rukbah, Dalal A. Alfawaz, Saad M. Wali

**Affiliations:** 1 Department of Pharmacology and Toxicology, Faculty of Medicine, Umm Al-Qura University, Al-Qunfudhah, Saudi Arabia; 2 College of Pharmacy, Umm Al-Qura University, Makkah, Saudi Arabia; 3 Department of Pharmacy Practice, Faculty of Pharmacy, University of Tabuk, Tabuk, Saudi Arabia; 4 Department of Clinical Pharmacology, Faculty of Medicine, King Abdulaziz University, Jeddah, Saudi Arabia; 5 Pharmacology and Toxicology Department, College of Pharmacy, Umm Al-Qura University, Makkah, Saudi Arabia

**Keywords:** KAP, knowledge, OTC medication, pharmacy students, self-medication, stress, university

## Abstract

**Background:**

Self-medication with over-the-counter (OTC) medicines is common among university students, particularly pharmacy students, despite the associated risks. Pharmacy students receive comprehensive education in pharmacology and medication safety. However, it remains unclear whether medication-related knowledge alone is sufficient to promote responsible self-medication practices or whether psychological factors, such as stress-related attitudes, also influence these behaviors. Addressing this gap is important, as pharmacy students are expected to promote the rational use of medicines in their future professional practice. Therefore, this study aimed to assess self-medication practices and associated factors among pharmacy students at Umm Al-Qura University in Makkah, Saudi Arabia.

**Methods:**

A cross-sectional study was conducted among full-time Doctor of Pharmacy (Pharm. D.) students at Umm Al-Qura University in Makkah, Saudi Arabia, between November and December 2025. Data were collected using a convenience sampling method through an electronic questionnaire developed by pharmacology faculty members. The questionnaire underwent review for face and content validity and was pilot-tested before administration. It assessed demographic characteristics, self-medication practices, medication-related knowledge, and stress-related attitudes. Descriptive statistics, chi-square tests or Fisher's exact tests, and Wilcoxon rank-sum tests were employed to analyze the study variables. Multivariable logistic regression was used to examine associations of demographic characteristics, medication-related knowledge, and stress-related attitudes with frequent self-medication. Statistical significance was defined as *p* < 0.05.

**Results:**

A total of 244 pharmacy students participated, including 161 (66.0%) females and 83 (34.0%) males. 230 students (94.3%) reported practicing self-medication. Among them, the most frequently used categories were painkillers (189, 82.2%), topical preparations (154, 67.0%), and cough syrups (106, 46.1%). Male students were more likely than females to report self-medication for financial and time-saving reasons and were also more likely to use cough syrup. In contrast, female students reported greater use of sleeping aids. Higher medication-related knowledge (OR=1.21, 95% CI: 1.12–1.32; *p* < 0.001) and stress-related attitudes (OR=1.11, 95% CI: 1.04–1.19; *p* = 0.004) were independently associated with frequent self-medication.

**Conclusion:**

Medication-related knowledge and stress-related attitudes emerged as significant independent predictors of frequent self-medication among pharmacy students. These findings indicate the importance of integrating medication safety education with stress management strategies in pharmacy curricula to promote responsible self-medication practices among future pharmacists. Implementing such interventions may facilitate the rational use of OTC medicines, enhance patient safety, and improve public health outcomes.

## Introduction

Self-medication is an aspect of self-care in which individuals play an active role in managing minor health conditions using non-prescription, over-the-counter (OTC) medicines. These medications are considered safe and effective when used according to directions. The World Health Organization (WHO) defines self-medication as the use of medicines by individuals to treat self-recognized illnesses or symptoms, including the intermittent or continued use of prescribed drugs for chronic or recurrent conditions beyond the recommended duration (1).

Healthcare professionals, especially community pharmacists, often support self-medication by providing advice and promoting the safe and rational use of medicines. Their involvement can enhance the management of minor illnesses and reduce unnecessary physician consultations. However, the reclassification of prescription-only medicines to OTC status has increased the availability and accessibility of self-medication products in pharmacies and retail settings worldwide (2, 3). The rise in self-medication is linked to several factors, including socioeconomic constraints such as limited income and time, easy access to medicines, fast-paced lifestyles, healthcare system inefficiencies, high healthcare costs, limited access to medical services, and inadequate regulation of medication distribution (4).

Responsible self-medication involves using OTC medicines with an established safety profile to treat self-recognized symptoms or minor conditions (5). When practiced correctly, it can prevent or relieve mild illnesses and reduce healthcare costs. However, safe self-medication requires consumers to interpret symptoms accurately, choose appropriate medications, follow recommended dosages and treatment durations, and consider their medical history, comorbidities, contraindications, and potential adverse effects. The WHO emphasizes that responsible self-medication relies on access to accurate health information and the rational use of medicines to maximize benefits and minimize risks (6).

Self-medication offers several potential benefits, such as reduced healthcare costs, fewer physician visits, shorter hospital waiting times, and more efficient allocation of healthcare resources. However, inappropriate self-medication may lead to adverse outcomes, including drug dependence, antimicrobial resistance, delayed diagnosis and treatment of serious conditions, masking of underlying symptoms, disease progression, drug interactions, and unintended adverse drug reactions (7–9). Additionally, the use of leftover medicines from previous treatments constitutes another form of self-medication and may increase the risk of inappropriate use (10).

Self-medication is a growing public health concern that has been studied across various populations and regions. Research among medical, pharmacy, and other healthcare students in Asia, Africa, and Europe has reported prevalence rates of 60% to 95% (11–16). Common reasons for self-medication include prior experience with similar symptoms, confidence in personal knowledge, limited time to consult a physician, and barriers such as cost and long waiting times (13, 17). In Saudi Arabia, studies have found a high prevalence of self-medication among students in medical, pharmacy, and health-related fields (17–22). Reported rates range from 50% to 90%, with analgesics, antipyretics, and non-opioid pain relievers being the most frequently used medications (17, 19–22). Pharmacies, previous prescriptions, and internet sources are common sources of medication information (17, 18, 20, 23).

Psychological stress is increasingly recognized as a significant factor influencing self-medication practices among university students, particularly those enrolled in health-related disciplines. Compared with students in other academic fields, health science students often experience greater academic pressure due to intensive coursework, frequent examinations, and demanding clinical and educational requirements. These factors may promote self-medication as a coping strategy for stress-related symptoms (24). In Saudi Arabia, more than half of pharmacy and medical students reported moderate levels of perceived stress with examinations, heavy course loads, and assignment workloads identified as principal stressors (25). Consistent with these findings, studies have demonstrated that stress is associated with more frequent use of analgesics, sedatives, and other non-prescription medications for rapid symptom relief (24). Among medical students in India, approximately 62% reported using non-prescribed medications to alleviate stress-related symptoms such as body pain and insomnia (26). Health-related stress has also been identified as an independent predictor of self-medication practices among undergraduate medical students, suggesting that higher stress levels may increase the likelihood of unsupervised medication use (24). These findings suggest that psychological stress may contribute not only to the initiation of self-medication but also to more frequent or potentially inappropriate medication use.

In addition to psychological stress, medication-related knowledge has been recognized as an important determinant of responsible self-medication (27). Adequate pharmacological knowledge may support appropriate medication selection, correct dosing, recognition of adverse effects, and timely consultation with healthcare professionals when necessary. Previous research indicates that enhancing medication-related knowledge through education and formal training may encourage more responsible self-medication practices among future healthcare professionals (28). Despite reports of satisfactory knowledge and generally positive attitudes toward self-medication, inappropriate practices remain common (12, 15, 16), including unsafe storage, misuse of leftover medications, and reliance on non-professional sources of information (17, 18, 21). Although pharmacological knowledge can support more informed decision-making, it may also increase confidence in self-diagnosis and self-treatment without professional supervision.

Although previous studies have examined the prevalence of self-medication and individual determinants such as stress or medication-related knowledge, there is limited evidence on their combined influence alongside demographic characteristics among pharmacy students, especially in Saudi Arabia. As future healthcare professionals with pharmacological training and greater access to medication-related information, pharmacy students are a key group for examining the determinants of self-medication and identifying ways to promote rational medication use. Such evidence can inform educational strategies, enhance pharmaceutical care, and support public health interventions to encourage responsible self-medication in Saudi Arabia. This study therefore aimed to assess self-medication practices among pharmacy students at Umm Al-Qura University and to examine the associations of medication-related knowledge, stress-related attitudes, demographic characteristics, and other relevant factors with frequent self-medication in Makkah, Saudi Arabia.

## Methods

### Study design

A cross-sectional study was conducted among Doctor of Pharmacy (Pharm. D.) students at Umm Al-Qura University (UQU), Makkah, Saudi Arabia, from November to December 2025. An electronic questionnaire was distributed to eligible students to examine self-medication practices and associated factors.

### Study setting and participants

All enrolled Pharm. D. students aged ≥18 years were eligible for inclusion. Students from all academic years were invited via the electronic communication system; two reminder messages were sent during data collection to increase participation. Participation was voluntary. Only UQU pharmacy students were eligible to participate, and incomplete questionnaires were excluded from the analysis.

### Survey instrument

The questionnaire was developed and reviewed by pharmacology faculty members to establish face and content validity. A pilot test was conducted among a small group of students (*n* = 7) to ensure clarity, relevance, and feasibility. Minor modifications were made based on participants’ feedback prior to final distribution. Although the pilot sample was small, it was sufficient to identify and resolve ambiguities in item phrasing and response options before administration of the full survey. The final survey was administered electronically, and all responses were collected anonymously. The full English version of the questionnaire is provided as a Supplementary Questionnaire. Internal consistency reliability was assessed using Cronbach's alpha. Confirmatory factor analysis (CFA) was performed to assess construct validity (Supplementary Table 2). The knowledge scale demonstrated excellent model fit (*χ*^2^ = 5.604, df = 5, *p* = 0.347; CFI = 0.998; RMSEA = 0.022), confirming unidimensionality. The stress-related attitudes scale showed marginal fit (*χ*^2^ = 18.077, *p* = 0.003; CFI = 0.941; RMSEA = 0.104). Both scales demonstrated acceptable internal consistency and were retained for analysis.

The questionnaire consisted of five sections. The first section collected demographic information, including gender, academic year, grade point average (GPA), and prior clinical or extracurricular training related to medications. The second section assessed medication-related knowledge, defined as understanding of appropriate medication selection, correct dosage, prescription requirements, potential side effects, contraindications, and situations requiring professional medical advice. The third section evaluated stress-related experiences and behaviors relevant to self-medication, such as academic stress, stress-related medication use, and coping behaviors. The fourth section assessed self-medication practices, defined as participants’ medication-related behaviors. These behaviors included the frequency of self-medication, motivations for self-medication, types of medications used, medication-checking behaviors, and occurrence of adverse effects. The final section explored participants’ perceptions of the relative influence of medication knowledge and stress-related attitudes on self-medication decisions. Knowledge and stress-related attitudes scores were calculated using five Likert-scale items from their respective domains.

### Scoring

Knowledge and stress-related attitudes scores were calculated by summing responses to their respective items (*n* = 5), each rated on a 5-point Likert scale (1 = strongly disagree to 5 = strongly agree). The internal consistency of both domains was assessed using Cronbach's alpha. The knowledge score ranged from 5 to 25, with higher scores indicating greater medication-related knowledge. The stress-related attitudes score also ranged from 5 to 25, with higher scores reflecting a greater negative impact of stress-related attitudes on medication safety and self-medication behaviors.

### Sample size calculation

Sample size was determined *a priori* using power analysis in R (version 4.6.0). Assuming *α* = 0.05, power of 0.90, and seven predictors (gender, academic year, GPA, training, healthcare professional in the household, knowledge, and stress-related attitudes), a medium effect size (f^2^ = 0.15) required a sample size of *n* ≥ 121. The final sample of *n* = 244 provided approximately 90% power to detect multivariable associations.

### Ethics

Ethical approval for this research was granted by the Scientific Biomedical Research Ethics Committee at the College of Medicine, Umm Al-Qura University (Approval Number: HAPO-02-K-012-2025-12-3083). The study was conducted in accordance with the Declaration of Helsinki and relevant national research ethics regulations. Participation was voluntary, and participants provided digital informed consent before completing the questionnaire. All responses were collected anonymously and treated confidentially.

### Statistical analysis

Data were analyzed using RStudio (version 2024.9.1.394, Boston, MA, USA) with R version 4.4.2. Descriptive statistics, including frequencies and percentages, summarized categorical variables, while medians and interquartile ranges (IQR) summarized continuous variables. Associations between categorical variables were assessed using Pearson's chi-square test or Fisher's exact test, as appropriate. The Wilcoxon rank-sum test was used to compare median knowledge and stress-related attitudes scores between groups.

To identify independent predictors of frequent self-medication, ordinal logistic regression was initially considered to preserve the five-category outcome (frequency of self-medication: Never, Rarely, Sometimes, Often, Always). Ordinal logistic regression models were fitted using the clm() function from the ordinal package. The proportional odds assumption, a key requirement for ordinal regression, was tested using the brant() function from the brant package via likelihood ratio testing. Both unadjusted and adjusted models violated this assumption (Model 1: *χ*^2^ = 18.549, df = 6, *p* = 0.005; Model 2: *χ*^2^ = 47.182, df = 30, *p* = 0.024), indicating heterogeneous covariate effects across outcome levels. Additionally, sparse cells were observed in the GPA < 2.00 category (*n* = 2); recoding GPA into three categories (< 3.00, 3.00–3.49, 3.50–4.00) partially addressed this sparseness, but violations of the assumption persisted.

Given these violations, the outcome variable was dichotomized as ‘Often/Always’ (habitual self-medication) vs. ‘Never/Rarely/Sometimes’ (non-habitual use) to facilitate valid statistical inference. This cut-off was defined as a study-specific analytical definition to distinguish students reporting frequent self-medication from those reporting occasional or infrequent self-medication. This dichotomization provided a clinically meaningful distinction between these groups for the subsequent regression analysis. A multivariable logistic regression model was then applied using the glm() function to identify independent predictors of habitual self-medication, with odds ratios (OR) and 95% confidence intervals (CI) reported. Multicollinearity was assessed using variance inflation factors (VIF) via the car package. The model included gender, academic year, GPA, medication-related training, healthcare professional in the household, knowledge score, and stress-related attitudes score as independent variables. A *p*-value of less than 0.05 was considered statistically significant throughout the analysis.

Ordinal logistic regression results are presented in Supplementary Tables 3, 4 for comparison. Despite violations of the proportional odds assumption, these supplementary analyses demonstrated comparable effect estimates for the primary predictors (knowledge and stress-related attitudes), supporting the robustness of the dichotomized approach.

## Results

### Demographic characteristics of pharmacy students

Of the 244 pharmacy students who participated in the study, 66.0% were female and 57.4% were in the intermediate academic years. Most students reported a GPA between 3.50 and 4.00 (48.4%). More than half had not received medication-related training outside the regular curriculum, and 45.5% reported having a family member or colleague working in a healthcare profession. Regarding self-medication frequency, about two-thirds of students reported self-medicating sometimes (37.3%) or often (27.5%) (Table 1).

**Table 1:** Demographic characteristics of the participants (*n* = 244).

Characteristic	Description
Gender	
Male	83 (34.0%)
Female	161 (66.0%)
Academic year	
Junior student (1st + 2nd year)	61 (25.0%)
Intermediate student (3rd + 4th year)	140 (57.4%)
Senior student (5th + 6th year)	43 (17.6%)
Current GPA	
<2.00	2 (0.8%)
2.00–2.99	27 (11.1%)
3.00–3.49	97 (39.8%)
3.50–4.00	118 (48.4%)
Ever received clinical or extracurricular training related to medications outside your regular coursework (e.g., summer training in a pharmacy or hospital)	
No	131 (53.7%)
Yes, once	72 (29.5%)
Yes, more than once	41 (16.8%)
Anyone living in your home or workplace is in a medical or healthcare profession	
No	133 (54.5%)
Yes	111 (45.5%)
Frequency of self-medication	
Never	14 (5.7%)
Rarely	37 (15.2%)
Sometimes	91 (37.3%)
Often	67 (27.5%)
Always	35 (14.3%)

*n*(%).

### Self-medication practice among pharmacy students

Among the 230 pharmacy students who reported self-medicating, the primary reason for self-medication was knowing which medication to take (67.4%), followed by prior experience with the medication (47.4%). Painkillers were the most frequently self-administered medications (82.2%). Approximately 39% of students always read medication labels or instructions, while 29% reported experiencing side effects related to self-medication (Table 2).

**Table 2:** Self-medication practices among pharmacy students (*n* = 230).

Characteristic	Description
Reason for self-medication^a^	
I believe the illness is minor	110 (47.8%)
I cannot afford a doctor’s visit	22 (9.6%)
I have prior experience with the medication	109 (47.4%)
I know which medication to take	155 (67.4%)
To save time	105 (45.7%)
Types of medications usually self-administered^a^	
Anxiolytic	25 (10.9%)
Cough syrup	106 (46.1%)
Topical creams/gels	154 (67.0%)
Sleeping aids	69 (30.0%)
Painkillers	189 (82.2%)
Frequency of reading medication labels or instructions when self- medicating	
Never	13 (5.7%)
Rarely	23 (10.0%)
Sometimes	59 (25.7%)
Often	46 (20.0%)
Always	89 (38.7%)
Ever experienced side effects as a result of self-medication	
No	163 (70.9%)
Yes	67 (29.1%)

a An asterisk indicates a multiple-response item *n* (%).

### Medication-related knowledge among pharmacy students

**Figure 1:**
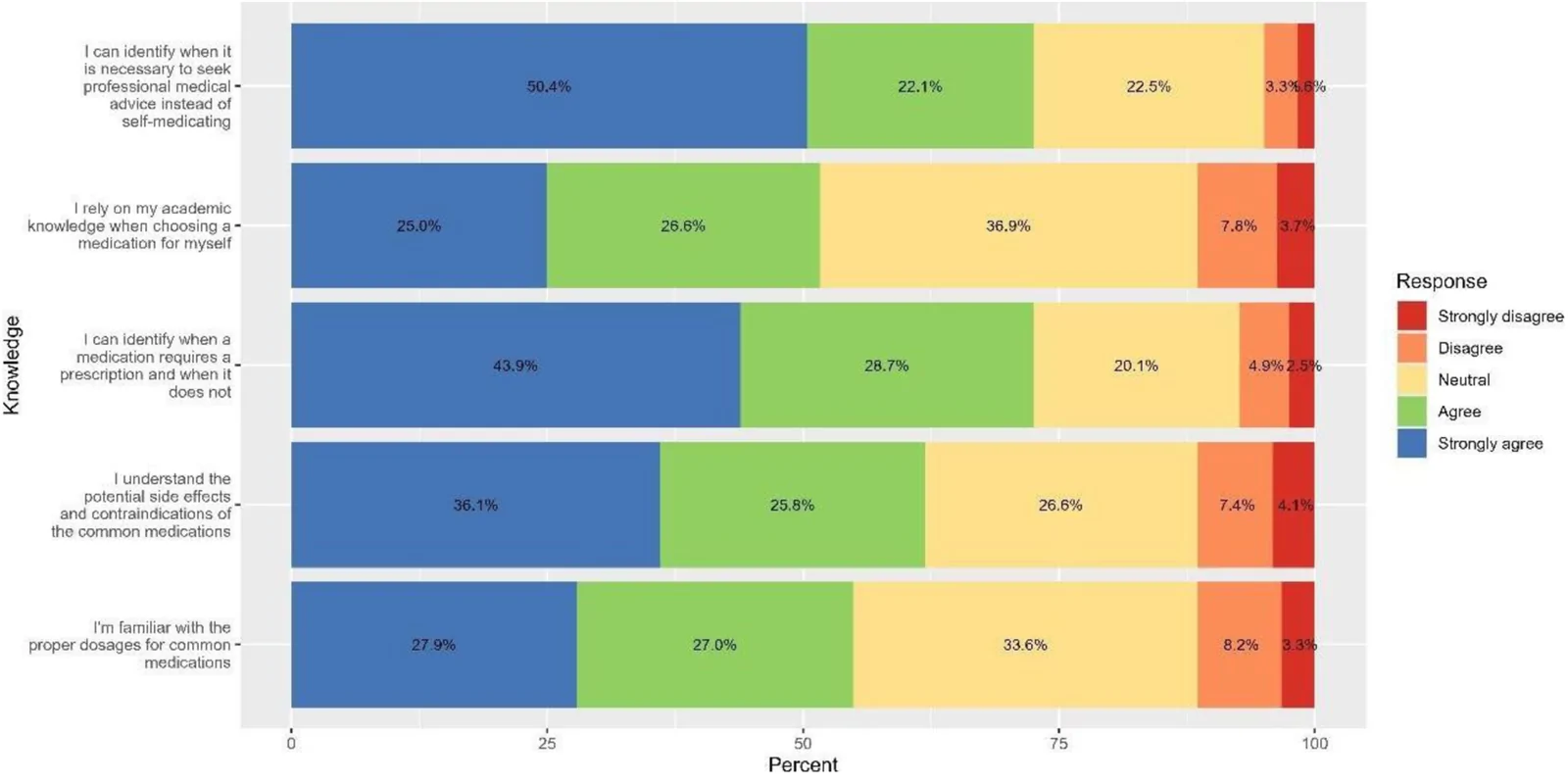
Participants’ responses to knowledge items.

Pharmacy students demonstrated strong medication-related knowledge. Most reported the ability to identify prescription-only medications (72.6%) and recognize situations requiring professional medical advice rather than self-medication (72.5%). In addition, 51.6% relied on academic knowledge when selecting medications and 54.9% were familiar with the proper dosages of common medications (Figure 1).

### Stress-related self-medication attitudes among pharmacy students

**Figure 2:**
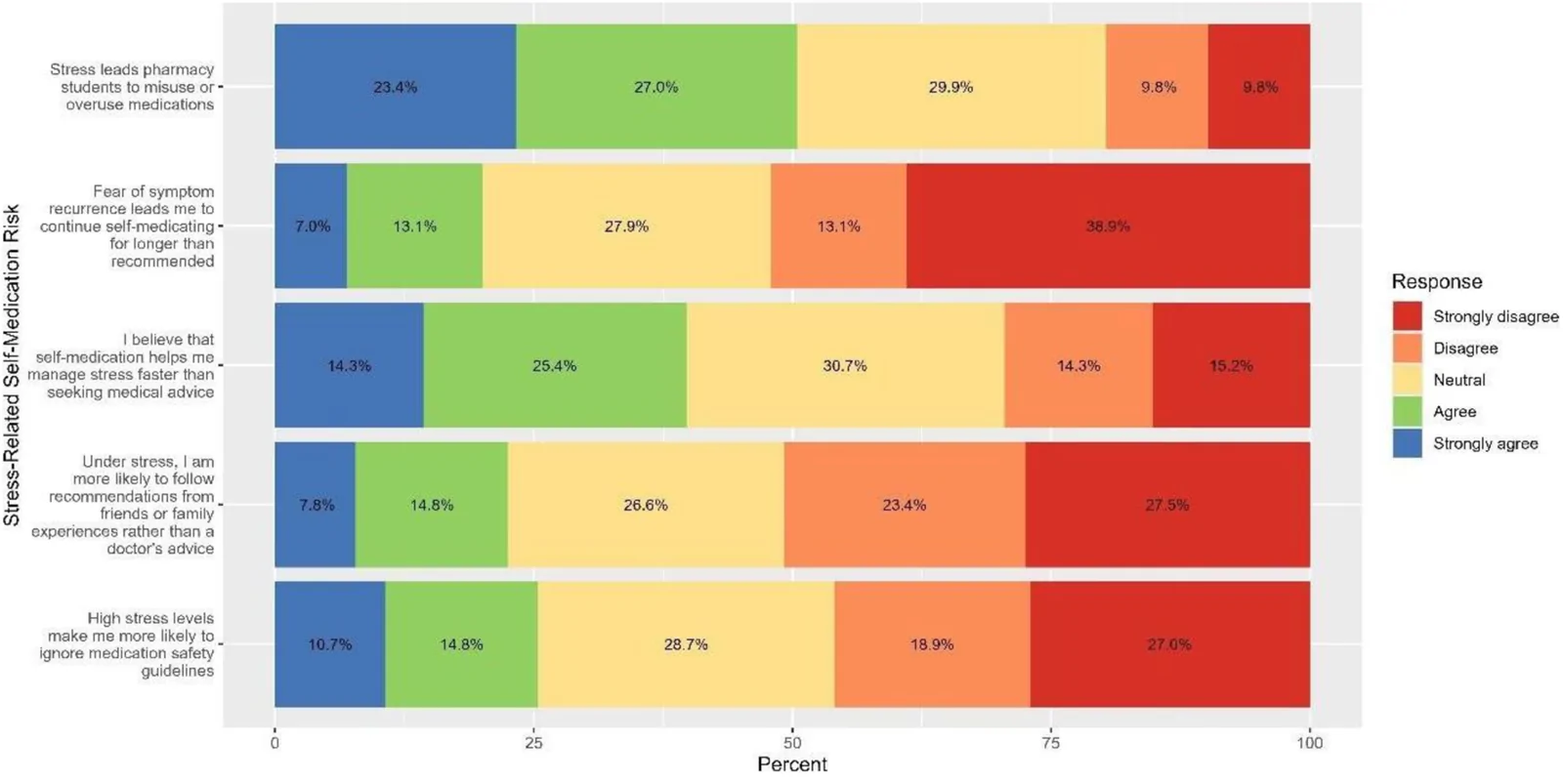
Participants’ responses to items assessing stress-related self-medication attitudes.

Responses to stress-related items suggested generally cautious attitudes toward self-medication under stress. Most students disagreed that stress encouraged unsafe medication practices (46.3%) or increased reliance on advice from friends or family rather than healthcare professionals (50.9%). Nevertheless, 39.7% believed that self-medication provided faster relief from stress-related symptoms, and 50.4% agreed that stress could contribute to medication misuse or overuse (Figure 2).

### Knowledge and stress-related attitudes scores

Knowledge and stress-related attitudes scores were assessed for normality using the Shapiro–Wilk test (*p* < 0.05 for both), indicating non-normal distributions. Consequently, scores were reported as medians (IQRs): knowledge, 19.5 (16.0–23.0, range 7.0–25.0) and stress-related attitudes, 14.0 (11.0–17.0, range 5.0–25.0). Internal consistency was acceptable: Cronbach's *α* = 0.782 for knowledge and *α* = 0.705 for stress-related attitudes (Table 3).

**Table 3:** Descriptive statistics for knowledge and stress scores among pharmacy students.

Characteristic	Median (Interquartile Range)	Min-Max	Cronbach Alpha	N of items	Shapiro *p*-value
Knowledge	19.5 (16.0–23.0)	7.0–25.0	0.782	5	<0.001
Stress	14.0 (11.0–17.0)	5.0–25.0	0.705	5	0.002

### Associations between demographic characteristics and self-medication practice

Several demographic characteristics were associated with self-medication practices. Compared with females, males were more likely to report self-medicating because they could not afford a doctor's visit (18.3% vs. 4.7%, *p* = 0.002) and to save time (58.5% vs. 38.5%, *p* = 0.005). Males were also more likely to use cough syrup (56.1% vs. 40.5%, *p* = 0.033) and to report experiencing side effects from self-medication (39.0% vs. 23.6%, *p* = 0.021).

Junior-year students were significantly more likely than intermediate- and senior-year students to report knowing which medication to take as a reason for self-medication (83.1% vs. 61.5% vs. 63.4%, *p* = 0.012). Students with higher GPAs reported more frequent use of sleeping aids (7.4% among those with a GPA < 3.00, 30.0% among those with a GPA of 3.00–3.49, and 35.4% among those with a GPA of 3.50–4.00, *p* = 0.017). Painkiller use increased from 59.3% among students with GPAs < 3.00 to approximately 85% among those with GPAs ≥ 3.00 (*p* = 0.009) (Table 4).

**Table 4:** Associations between demographic characteristics and self-medication practices (*n* = 230).

	Gender				Academic year					GPA				
Characteristic	Male *N* = 82	Female *N* = 148	Chi- Square	*p*- value	Junior student *N* = 59	Intermediate student *N* = 130	Senior student *N* = 41	Chi- Square	*p*- value	<2.00 - 2.99 *N* = 27	3.00–3.49 *N* = 90	3.50–4.00 *N* = 113	Chi- Square	*p*- value
Reason for self-medication^a^														
I believe the illness is minor	41 (50.0%)	69 (46.6%)	0.12	0.724	32 (54.2%)	57 (43.8%)	21 (51.2%)	2.0	0.370	12 (44.4%)	43 (47.8%)	55 (48.7%)	0.16	0.925
I cannot afford a doctor’s visit	15 (18.3%)	7 (4.7%)	9.7	0.002	4 (6.8%)	16 (12.3%)	2 (4.9%)	2.7	0.259	2 (7.4%)	10 (11.1%)	10 (8.8%)	0.46	0.794
I have prior experience with the medication	35 (42.7%)	74 (50.0%)	0.86	0.354	30 (50.8%)	57 (43.8%)	22 (53.7%)	1.6	0.453	9 (33.3%)	49 (54.4%)	51 (45.1%)	4.2	0.124
I know which medication to take	55 (67.1%)	100 (67.6%)	0.00	>0.999	49 (83.1%)	80 (61.5%)	26 (63.4%)	8.9	0.012	15 (55.6%)	61 (67.8%)	79 (69.9%)	2.1	0.358
To save time	48 (58.5%)	57 (38.5%)	7.7	0.005	26 (44.1%)	57 (43.8%)	22 (53.7%)	1.3	0.525	15 (55.6%)	44 (48.9%)	46 (40.7%)	2.6	0.278
Types of medications usually self-administered^a^														
Anxiolytic	13 (15.9%)	12 (8.1%)	2.5	0.113	5 (8.5%)	16 (12.3%)	4 (9.8%)	0.68	0.712	3 (11.1%)	12 (13.3%)	10 (8.8%)	1.0	0.594
Cough syrup	46 (56.1%)	60 (40.5%)	4.5	0.033	29 (49.2%)	58 (44.6%)	19 (46.3%)	0.34	0.845	12 (44.4%)	48 (53.3%)	46 (40.7%)	3.2	0.197
Topical creams/gels	53 (64.6%)	101 (68.2%)	0.17	0.681	40 (67.8%)	89 (68.5%)	25 (61.0%)	0.81	0.665	17 (63.0%)	63 (70.0%)	74 (65.5%)	0.68	0.711
Sleeping aids	18 (22.0%)	51 (34.5%)	3.4	0.067	12 (20.3%)	42 (32.3%)	15 (36.6%)	3.8	0.150	2 (7.4%)	27 (30.0%)	40 (35.4%)	8.1	0.017
Painkillers	66 (80.5%)	123 (83.1%)	0.10	0.751	52 (88.1%)	106 (81.5%)	31 (75.6%)	2.7	0.263	16 (59.3%)	77 (85.6%)	96 (85.0%)	11	0.004
Frequency of reading medication labels or instructions when self- medicating			6.9	0.143				5.3	0.728				5.6	0.689
Never	5 (6.1%)	8 (5.4%)			2 (3.4%)	8 (6.2%)	3 (7.3%)			0 (0.0%)	4 (4.4%)	9 (8.0%)		
Rarely	5 (6.1%)	18 (12.2%)			8 (13.6%)	12 (9.2%)	3 (7.3%)			1 (3.7%)	10 (11.1%)	12 (10.6%)		
Sometimes	27 (32.9%)	32 (21.6%)			10 (16.9%)	38 (29.2%)	11 (26.8%)			9 (33.3%)	21 (23.3%)	29 (25.7%)		
Often	19 (23.2%)	27 (18.2%)			13 (22.0%)	24 (18.5%)	9 (22.0%)			6 (22.2%)	17 (18.9%)	23 (20.4%)		
Always	26 (31.7%)	63 (42.6%)			26 (44.1%)	48 (36.9%)	15 (36.6%)			11 (40.7%)	38 (42.2%)	40 (35.4%)		
Ever experienced side effects as a result of self- medication			5.3	0.021				0.40	0.817				3.0	0.227
No	50 (61.0%)	113 (76.4%)			40 (67.8%)	93 (71.5%)	30 (73.2%)			18 (66.7%)	59 (65.6%)	86 (76.1%)		
Yes	32 (39.0%)	35 (23.6%)			19 (32.2%)	37 (28.5%)	11 (26.8%)			9 (33.3%)	31 (34.4%)	27 (23.9%)		

a An asterisk indicates a multiple-response item; *χ*² or Fisher’s exact test.

n (%).

Pearson’s Chi-squared test.

### Factors associated with frequent self-medication

Knowledge and stress-related attitudes scores differed significantly between students with frequent and non-frequent self-medication. The knowledge score was higher among students with frequent self-medication (median 21.0, IQR 19.0–24.0) than among students with non-frequent self-medication (median 18.0, IQR 15.0–21.0, *p* < 0.001). The stress-related attitudes score was also higher among students with frequent self-medication (median 15.0, IQR 11.0–18.0) than among students with non-frequent self-medication (median 14.0, IQR 9.0–16.0, *p* = 0.007) (Table 5).

**Table 5:** Factors associated with self-medication practice.

Characteristic	Never to Sometimes *N* = 142	Often/Always *N* = 102	Chi- Square	*p*- value
Gender			0.04	0.849
Male	49 (59.0%)	34 (41.0%)		
Female	93 (57.8%)	68 (42.2%)		
Academic year			1.6	0.443
Junior student (1st + 2nd year)	34 (55.7%)	27 (44.3%)		
Intermediate student (3rd + 4th year)	86 (61.4%)	54 (38.6%)		
Senior student (5th + 6th year)	22 (51.2%)	21 (48.8%)		
Current GPA				0.766
<2.00	1 (50.0%)	1 (50.0%)		
2.00–2.99	18 (66.7%)	9 (33.3%)		
3.00–3.49	54 (55.7%)	43 (44.3%)		
3.50–4.00	69 (58.5%)	49 (41.5%)		
Ever received clinical or extracurricular training related to medications outside your regular coursework (e.g., summer training in a pharmacy or hospital)			3.9	0.146
No	76 (58.0%)	55 (42.0%)		
Yes, once	47 (65.3%)	25 (34.7%)		
Yes, more than once	19 (46.3%)	22 (53.7%)		
Anyone living in your home or workplace is in a medical or healthcare profession	64 (57.7%)	47 (42.3%)	0.02	0.876
Knowledge score	18.0 (15.0–21.0)	21.0 (19.0–24.0)	4,492	<0.001
Stress score	14.0 (9.0–16.0)	15.0 (11.0–18.0)	5,782	0.007

Pearson’s Chi-squared test; Fisher’s exact test; Wilcoxon rank sum test.

*n*(%); Median (Interquartile Range).

*χ*², chi-square test statistic; Wilcoxon rank-sum test statistic reported for continuous variables (knowledge and stress scores).

### Predictors of frequent self-medication

In the adjusted multivariable logistic regression model, higher medication-related knowledge (OR=1.21, 95% CI 1.12–1.32, *p* < 0.001) and higher stress-related attitudes scores (OR=1.11, 95% CI 1.04–1.19, *p* = 0.004) were significantly associated with frequent self-medication. None of the demographic variables, including gender, academic year, GPA, medication-related training, or having a healthcare professional in the household, showed statistically significant associations (Table 6).

**Table 6:** Predictors of self-medication practice.

		Model 1			Model 2	
Characteristic	OR	95% CI	*p*-value	OR	95% CI	*p*-value
Knowledge score	1.21	1.12, 1.32	<0.001	1.21	1.12, 1.32	<0.001
Stress score	1.10	1.03, 1.18	0.004	1.11	1.04, 1.19	0.004
Gender						
Male	NA	NA	NA	Reference	Reference	
Female	NA	NA	NA	1.05	0.54, 2.05	0.883
Academic year						
Junior student (1st + 2nd year)	NA	NA	NA	Reference	Reference	
Intermediate student (3rd + 4th year)	NA	NA	NA	0.87	0.44, 1.73	0.680
Senior student (5th + 6th year)	NA	NA	NA	1.63	0.67, 3.99	0.282
Current GPA						
<2.00	NA	NA	NA	Reference	Reference	
2.00–2.99	NA	NA	NA	0.36	0.01, 10.5	0.501
3.00–3.49	NA	NA	NA	0.49	0.02, 13.2	0.622
3.50–4.00	NA	NA	NA	0.48	0.02, 13.1	0.618
Ever received training related to medications						
No	NA	NA	NA	Reference	Reference	
Yes, once	NA	NA	NA	0.65	0.34, 1.24	0.199
Yes, more than once	NA	NA	NA	1.22	0.55, 2.76	0.626
Anyone living in your home or workplace is in a medical or healthcare profession	NA	NA	NA			

CI, confidence interval; OR, odds ratio.

Model 1: Unadjusted model including knowledge and stress scores only. Model 2: Adjusted model including knowledge, stress, and demographic variables (gender, academic year, GPA, prior medication training, healthcare family contact).

## Discussion

This study examined self-medication practices among pharmacy students and found a high prevalence of self-medication, including among students with strong academic performance. Medication-related knowledge and stress-related attitudes emerged as independent predictors of frequent self-medication, whereas demographic characteristics were not independently associated with frequent self-medication practice. These results suggest that knowledge alone may be insufficient to ensure safe self-medication practices and highlight the need to consider psychological and behavioral factors.

Self-medication was prevalent among pharmacy students in the present study, consistent with previous reports from Saudi Arabia and other countries that identify self-medication as a widespread practice among university students, particularly those enrolled in health-related disciplines. This high prevalence is likely attributed to students’ familiarity with common medications, confidence in managing minor illnesses, and easy access to OTC medicines. A meta-analysis of 89 studies involving 60,938 students found that approximately 70% of university students worldwide engage in self-medication, with higher rates among medical and pharmacy students compared to those in non-health disciplines (29). Studies from Saudi Arabia have similarly reported a high prevalence of self-medication among medical and pharmacy students, further supporting the findings of the present study (18, 19, 21, 22, 30–32). These findings suggest that self-medication is an established health behavior among healthcare students, likely influenced by early exposure to medication-related knowledge and increased confidence in independently managing minor health conditions. This underscores the importance of promoting safe and responsible self-medication practices (24).

Analysis of demographic characteristics revealed that male students were more likely than female students to report self-medication for reasons related to time-saving and cost. One possible explanation is that males may be less likely to seek professional healthcare for minor illnesses and may prioritize convenience, resulting in greater reliance on self-medication. Convenience and cost have been consistently identified as common motivations for self-medication among university students (22). However, previous research demonstrates that self-medication behaviors differ between men and women, with the direction and magnitude of these differences varying across populations. For instance, studies involving healthcare students have reported higher self-medication rates among female students, primarily due to the use of analgesics for menstrual pain (28). In contrast, a large European population-based study found that self-medication patterns varied by sex and were influenced by pain severity, educational level, and barriers to healthcare access (33). Collectively, these findings indicate that biological, educational, and healthcare-related factors may contribute to gender differences in self-medication.

The present study found that pharmacy students generally possessed substantial medication-related knowledge, including appropriate medication selection, prescription requirements, potential adverse effects, and the importance of seeking professional medical advice. These results align with previous research among pharmacy students in Saudi Arabia, which also reported high levels of medication-related knowledge and awareness of the safe use of OTC medications (30, 31). This outcome likely reflects the strong emphasis on pharmacology, therapeutics, patient counselling, and evidence-based medication use within pharmacy curricula, as well as students’ exposure to clinical and community pharmacy training. Despite this strong knowledge base, inappropriate self-medication practices, such as antibiotic misuse and reliance on leftover medications, were still observed (30). This suggests that knowledge alone may not be sufficient to ensure safe self-medication and highlights the need for educational strategies that help translate knowledge into responsible self-medication practices.

This study also identified a significant association between stress-related attitudes and self-medication behaviors among pharmacy students. Stress may increase reliance on self-medication by prompting students to seek rapid relief from symptoms such as headaches, sleep disturbances, and anxiety, sometimes without adequate consideration of medication safety. Previous research similarly identified stress as a psychological factor associated with increased self-medication, particularly the use of analgesics and other frequently used medications (34, 35). In addition, stress has been shown to affect health-related decision-making, leading individuals to prioritize immediate symptom relief over appropriate medication use (36). These findings indicate that medication safety education alone may not be sufficient to promote responsible self-medication. Incorporating stress management strategies, resilience training, and behavioral support into pharmacy curricula may enable students to apply their medication knowledge more effectively during periods of academic or psychological stress (37, 38).

The multivariable analysis demonstrated that medication-related knowledge and stress-related attitudes were independent predictors of frequent self-medication. In contrast, demographic characteristics and academic achievement were not independently associated with this behavior after adjustment for potential confounders. Although academic achievement was associated with certain self-medication behaviors in the univariate analysis, it did not remain an independent predictor of frequent self-medication following multivariable adjustment.

The association between higher medication-related knowledge and more frequent self-medication may reflect greater confidence in recognizing symptoms and selecting appropriate medications, which can lead students to manage minor illnesses independently rather than seeking professional advice. However, this confidence may also contribute to overestimating one's ability to self-manage health conditions, potentially increasing reliance on self-medication. Likewise, stress-related attitudes may be associated with a greater tendency to seek immediate symptom relief at the expense of medication safety. Overall, these findings suggest that self-medication behavior is influenced by both cognitive factors, such as medication-related knowledge, and psychological factors related to stress. Therefore, educational strategies should extend beyond improving pharmacological knowledge and incorporate behavioral interventions, stress management, and medication safety training to encourage responsible self-medication practices.

### Study strengths

This study demonstrates several strengths. It included pharmacy students from all academic years, ensuring broad representation of the target population within the university. The sample size was determined using an *a priori* power calculation, thereby strengthening the statistical validity of the findings. The questionnaire was reviewed for face and content validity, pilot-tested before administration, and demonstrated acceptable internal consistency for both the knowledge and stress-related attitudes scales. In addition to describing the prevalence and patterns of self-medication, the study examined medication-related knowledge, stress-related attitudes, and demographic characteristics, and assessed their independent associations with frequent self-medication using multivariable logistic regression. These findings contribute to understanding the determinants of self-medication and provide evidence to inform pharmacy education, medication safety initiatives, and public health interventions.

### Study limitations

Several limitations should be considered when interpreting these findings. First, the study was conducted among pharmacy students at a single university, which may limit generalizability to students from other institutions and may not fully reflect self-medication practices among pharmacy students across Saudi Arabia. Nevertheless, students were recruited from all academic years, providing representation across different stages of pharmacy education, and the sample size exceeded the minimum required, enhancing the robustness of the statistical analyses. Future multicenter studies involving universities from various regions of Saudi Arabia are recommended to improve external validity and generalizability.

Second, the use of convenience sampling may have introduced selection bias and limited the study population's representativeness. In addition, reliance on self-reported questionnaires may have resulted in recall and social desirability biases. To minimize these potential sources of bias, the questionnaire was standardized, pilot-tested before implementation, administered anonymously, and completed voluntarily without collecting identifiable information. These measures likely encouraged honest responses and reduced response bias.

Third, the cross-sectional design precludes drawing conclusions about causal relationships between academic achievement, stress-related attitudes, and self-medication practices. Longitudinal studies are necessary to better understand the temporal relationships among these factors.

The study was limited to pharmacy students, who generally have greater exposure to pharmacological education and pharmaceutical information than students in other health and non-health disciplines. As a result, their self-medication behaviors may not be representative of the broader student population or the general public, which restricts the generalizability of these findings. Future research should include students from multiple academic disciplines and members of the general population to enable comparisons across educational backgrounds and enhance generalizability. Qualitative approaches, such as in-depth interviews or focus groups, could provide deeper insight into the motivations and contextual factors underlying self-medication practices.

## Conclusion

Medication-related knowledge and stress-related attitudes were identified as significant independent predictors of frequent self-medication among pharmacy students. Higher knowledge scores were associated with a greater likelihood of frequent self-medication, indicating that increased familiarity with medications may encourage independent treatment decisions without professional consultation. Similarly, higher stress-related attitudes scores were associated with a greater likelihood of frequent self-medication, suggesting an association between stress-related attitudes and medication-related behaviors. Despite their academic background, pharmacy students demonstrated inconsistencies in safe medication practices, such as irregular reading of medication instructions. Conversely, demographic characteristics were not independently associated with frequent self-medication.

These findings suggest the importance of addressing both cognitive and behavioral determinants of self-medication within pharmacy education. Enhancing medication safety education alongside stress management strategies may promote more responsible self-medication practices and safer use of OTC medicines, with potential benefits for patient safety and public health. However, the interpretation of these results should consider the study’s cross-sectional design and single-university context, which limit causal inference and generalizability.

## Recommendations

The findings of this study indicate that pharmacy education should prioritize responsible self-medication and medication safety by integrating behavioral and stress-management components into the curriculum alongside pharmacological instruction. Universities are also encouraged to implement educational initiatives that promote the appropriate use of OTC medicines, recognition of situations requiring professional medical advice, and awareness of the risks associated with inappropriate self-medication. These measures may help future pharmacists make informed decisions and educate patients effectively about the safe use of OTC medicines. Finally, multicenter longitudinal studies are needed to confirm these findings and evaluate the effectiveness of educational and behavioral interventions in promoting responsible and safe self-medication practices among pharmacy students.

## Data Availability

The original contributions presented in the study are included in the article/Supplementary Material, further inquiries can be directed to the corresponding author.
