# Supplementary material for: Influence of stress and knowledge on self-medication practices among pharmacy students: a cross-sectional study
Source: Front Med (Lausanne). 2026 Sep 11;13:1877740. doi: 10.3389/fmed.2026.1877740 (PMC13612264; doi:10.3389/fmed.2026.1877740)
Supplement: Supplementary file 1 [file Datasheet1.pdf]

Table S1: Questionnaire Items and Response Options

Domain	Question	Response Options
Demographic characteristics	Gender	Male; Female
	Academic year	Junior student (1st–2nd year); Intermediate student (3rd–4th year); Senior student (5th–6th year)
	Current GPA	<2.00; 2.00–2.99; 3.00–3.49; 3.50–4.00
	Ever received clinical or extracurricular training related to medications	No; Yes, once; Yes, more than once
	Anyone in household/workplace in healthcare profession	No; Yes
	Frequency of self-medication	Never; Rarely; Sometimes; Often; Always
Self-medication practice	Reason for self-medication (multiple response)*	I believe the illness is minor; I cannot afford a doctor's visit; I have prior experience with the medication; I know which medication to take; To save time
	Types of medications usually self-administered (multiple response)*	Anxiolytic; Cough syrup; Topical creams/gels; Sleeping aids; Painkillers
	Frequency of reading medication labels/instructions	Never; Rarely; Sometimes; Often; Always
	Ever experienced side effects from self-medication	No; Yes
Medication Knowledge	I'm familiar with the proper dosages for common medications	Strongly disagree; Disagree; Neutral; Agree; Strongly agree
	I understand the potential side effects and contraindications of the common medications	Strongly disagree; Disagree; Neutral; Agree; Strongly agree
	I can identify when a medication requires a prescription and when it does not	Strongly disagree; Disagree; Neutral; Agree; Strongly agree
	I rely on my academic knowledge when choosing a medication for myself	Strongly disagree; Disagree; Neutral; Agree; Strongly agree
	I can identify when it is necessary to seek professional medical advice instead of self-medicating	Strongly disagree; Disagree; Neutral; Agree; Strongly agree
Stress-related self-medication attitudes	High stress levels make me more likely to ignore medication safety guidelines	Strongly disagree; Disagree; Neutral; Agree; Strongly agree
	Under stress, I am more likely to follow recommendations from friends or family experiences rather than a doctor's advice	Strongly disagree; Disagree; Neutral; Agree; Strongly agree
	I believe that self-medication helps me manage stress faster than seeking medical advice	Strongly disagree; Disagree; Neutral; Agree; Strongly agree
	Fear of symptom recurrence leads me to continue self-medicating for longer than recommended	Strongly disagree; Disagree; Neutral; Agree; Strongly agree
	Stress leads pharmacy students to misuse or overuse medications	Strongly disagree; Disagree; Neutral; Agree; Strongly agree

**Multiple-response items; respondents could select more than one option.*

Table S2: Confirmatory Factor Analysis Results for Knowledge and Stress Scales

Fit Index	Knowledge	Stress-related self-medication attitudes
χ^2 (df)	5.604 (5)	18.077 (5)
p-value	0.347	0.003
CFI	0.998	0.941
TLI	0.996	0.881
RMSEA (90% CI)	0.022 (0.000–0.094)	0.104 (0.055–0.157)
SRMR	0.022	0.045
Item Loadings		
Item 1	0.617	0.745
Item 2	0.723	0.628
Item 3	0.626	0.492
Item 4	0.575	0.678
Item 5	0.699	0.429

Table S3: Factors associated with self-medication practice

Characteristic	Never<br>N=14	Rarely<br>N=37	Sometimes<br>N=91	Often<br>N=67	Always<br>N=35	p-value
Knowledge score	18.5 (15.0 - 22.0)	19.0 (16.0 - 22.0)	18.0 (15.0 - 21.0)	21.0 (18.0 - 24.0)	21.0 (20.0 - 24.0)	<0.001
Stress-related self-medication attitudes score	12.0 (9.0 - 16.0)	12.0 (8.0 - 14.0)	15.0 (11.0 - 17.0)	15.0 (12.0 - 18.0)	14.0 (11.0 - 18.0)	<0.001
Gender						0.041
Male	1 (1.2%)	9 (10.8%)	39 (47.0%)	24 (28.9%)	10 (12.0%)	
Female	13 (8.1%)	28 (17.4%)	52 (32.3%)	43 (26.7%)	25 (15.5%)	
Academic year						0.465
Junior student (1st + 2nd year)	2 (3.3%)	7 (11.5%)	25 (41.0%)	21 (34.4%)	6 (9.8%)	
Intermediate student (3rd + 4th year)	10 (7.1%)	22 (15.7%)	54 (38.6%)	35 (25.0%)	19 (13.6%)	
Senior student (5th + 6th year)	2 (4.7%)	8 (18.6%)	12 (27.9%)	11 (25.6%)	10 (23.3%)	
Current GPA						0.181
<2.00	0 (0.0%)	1 (50.0%)	0 (0.0%)	0 (0.0%)	1 (50.0%)	
2.00 - 2.99	2 (7.4%)	8 (29.6%)	8 (29.6%)	7 (25.9%)	2 (7.4%)	
3.00 - 3.49	7 (7.2%)	11 (11.3%)	36 (37.1%)	32 (33.0%)	11 (11.3%)	
3.50 - 4.00	5 (4.2%)	17 (14.4%)	47 (39.8%)	28 (23.7%)	21 (17.8%)	
Ever received clinical or extracurricular training related to medications						0.489
No	6 (4.6%)	22 (16.8%)	48 (36.6%)	36 (27.5%)	19 (14.5%)	
Yes, once	7 (9.7%)	9 (12.5%)	31 (43.1%)	15 (20.8%)	10 (13.9%)	
Yes, more than once	1 (2.4%)	6 (14.6%)	12 (29.3%)	16 (39.0%)	6 (14.6%)	
Anyone living in your home or workplace is in a medical or healthcare profession	5 (4.5%)	14 (12.6%)	45 (40.5%)	31 (27.9%)	16 (14.4%)	0.735

n (%); Median (Q1 - Q3)

Fisher's exact test; Pearson's Chi-squared test; Kruskal-Wallis rank sum test

Table S4: Predictors of self-medication practice based on the self-medication practice variable as a dependent variable (Likert-scale variable)

Characteristic	Model 1			Model 2		
	O8R	95% CI	p-value	OR	95% CI	p-value
Knowledge score	1.16	1.09, 1.24	<0.001	1.17	1.09, 1.25	<0.001
Stress-related self-medication attitudes	1.10	1.04, 1.17	<0.001	1.11	1.04, 1.17	<0.001
Gender						
Male	NA	NA	NA	Reference	Reference	
Female	NA	NA	NA	0.73	0.43, 1.23	0.238
Academic year						
Junior student (1st + 2nd year)	NA	NA	NA	Reference	Reference	
Intermediate student (3rd + 4th year)	NA	NA	NA	0.90	0.52, 1.57	0.710
Senior student (5th + 6th year)	NA	NA	NA	1.57	0.75, 3.32	0.231
Current GPA						
<3.00	NA	NA	NA	Reference	Reference	
3.00 - 3.49	NA	NA	NA	1.55	0.69, 3.49	0.285
3.50 - 4.00	NA	NA	NA	1.93	0.87, 4.33	0.107
Ever received clinical or extracurricular training related to medications outside your regular coursework (eg, summer training in a pharmacy or hospital)						
No	NA	NA	NA	Reference	Reference	
Yes, once	NA	NA	NA	0.74	0.43, 1.27	0.280
Yes, more than once	NA	NA	NA	0.98	0.50, 1.91	0.943
Anyone living in your home or workplace is in a medical or healthcare profession						
No	NA	NA	NA	Reference	Reference	
Yes	NA	NA	NA	1.15	0.72, 1.84	0.550

Model 1: Unadjusted model including knowledge and stress-related self-medication attitudes scores only. Model 2: Adjusted model including knowledge, stress-related attitudes, and demographic variables (gender, academic year, GPA, prior medication training, healthcare family contact).

Model 1: The analysis is based on a multivariable ordinal logistic regression model. Results of proportional odds assumption (χ^2 = 18.549, df = 6, p = 0.005). Multicollinearity assessment indicated that no variables had VIF > 5.

Model 2: The analysis is based on a multivariable ordinal logistic regression model. Results of proportional odds assumption (χ^2 = 47.182, df = 30, p = 0.024). Multicollinearity assessment indicated that no variables had VIF > 5.

Abbreviations: CI = Confidence Interval, OR = Odds Ratio
