# Supplementary material for: Influence of stress and knowledge on self-medication practices among pharmacy students: a cross-sectional study
Source: Front Med (Lausanne). 2026 Sep 11;13:1877740. doi: 10.3389/fmed.2026.1877740 (PMC13612264; doi:10.3389/fmed.2026.1877740)
Supplement: Supplementary file 2 [file Datasheet2.pdf]

<u>Is self-medication common among pharmacy students and is it influenced more by stress or access to knowledge</u>

You are invited to participate in a short study about self-medication among pharmacy students. The purpose of this survey is to measure how self-medication is common in pharmacy students and how much it effected by stress or by the knowledge the students have. It takes between 3-4 minutes. Your responses are anonymous and voluntary.

1- Do you consent to participate in this survey?

-yes

-No

Section I: Demographics

This part is designed to gather essential details about participants.

2- Gender:

-Male

-Female

3- You are a pharmacy student in Umm Al-Qura University?

-Yes, I'm

-No, I'm not

4-Does anyone living in your home work in a medical or healthcare profession?

-Yes

-No

5- Have you ever received clinical or extracurricular training related to medications outside your regular coursework? (e.g., summer training in a pharmacy or hospital)

- Yes, more than once
- Yes, once
- No

6- Academic year:

-1st year

-2nd year

-3rd year

-4th year

-5th year

-6th year

Section 2: Knowledge

This section evaluates how much knowledge students currently have to help understanding their proficiency.

7- I'm familiar with the proper dosages for common over-the-counter (OTC) medications.

-Likert scale

8- I understand the potential side effects and contraindications of the common medications.

-Likert scale

9- I can identify when a drug requires a prescription and when it doesn't.

-Likert scale

10- I rely on my academic knowledge when choosing a medication for myself.

-Likert scale

11- I Can identify when it's necessary to seek professional medical advice instead of self-medicating.

-Likert scale

Section 3: Stress

This section evaluates the level of stress students experience and its impact on their self-medicated habits.

12- In the last month, how often have you felt overwhelmed by your responsibilities?

-Likert scale

13- I feel stressed during exams or high academic pressure.

-Likert scale

14- I tend to self-medicate more when I'm stressed.

-Likert scale

15- I use medications to manage headaches, insomnia or anxiety during stressful periods.

-Likert scale

16- Have you ever used medications (prescribed or not) just to cope with your stress?

-Yes, I have.

-No, I haven’t.

- No, but I've thought about doing it.

Section 4: Self-medication practice

This section looks at how common self-medication is among pharmacy students.

17- Have you taken any medication without a doctor's prescription in the past two months?

-Yes

-No

18- What kinds of medications do you usually self-medicate with?

-Painkillers

-anxiolytic

-sleeping aids

-Cough syrup

-topical creams/gels

19- How often do you self-medicate?

-Likert scale

20- Why do you self-medicate?

-I know what to take

-To save time

-I believe the illness is minor

-I have prior experience with the medication

-Can't afford a doctor

21- Do you usually check expiry dates, doses and side effects before taking a medication on your own?

-Likert chart

Section 5: Influence of knowledge vs stress

This section measures both stress and knowledge to understand how each one of them or both can affect self-medication behavior.

22- In your opinion, what influences your self-medication habits more?

-My knowledge as a pharmacy student

-My stress level

-Both equally

-Neither

23- My level of knowledge always guides me to make safe self-medication choices.

-Likert scale

24- High stress levels make me ignore my knowledge about medication safety.

-Likert scale

25- If pharmacy knowledge weren't available to you, would you still self-medicate?

-Yes

-No

26- Do you believe stress leads pharmacy students to misuse or overuse medications?

-Likert scale

<u>Confirmation message</u>

Thank you for completing this survey. Your participation contributes to a better understanding of student health behavior and may help improve pharmacy education and student support systems.
